# Private Medical Instruction

**Published:** 1842-08

**Authors:** 


### PRIVATE MEDICAL INSTRUCTION.

We owe an apology to Drs. Warder and Dennis, of Cincinnati, for having neglected to notice their circular, received some time since, announcing that they have associated themselves for the purpose of giving instruction in the various departments of Medicine and Surgery. The term commences on the first of March and continues one year; the mode of instruction will consist of daily examinations, interspersed with lectures and familiar demonstrations. During the winter especial reference is had to the current lectures in the Medical College of Ohio. From an intimate knowledge of these gentlemen, we do not hesitate to recommend them to favorable notice. The former is professor of Chemistry in Cincinnati College, and both are enthusiastically devoted to their profession. The course of study proposed, though a very extended one, will be faithfully carried out by them. C.

